# Leveraging Youth Sports to Deliver a Physical Activity Intervention to Mothers: Feasibility, Acceptability, and Preliminary Efficacy of a Single-Arm Open Pilot Trial

**DOI:** 10.1016/j.focus.2025.100465

**Published:** 2025-11-05

**Authors:** Tayla von Ash, Belinda O’Hagan, Sugandha K. Gupta-Louis, Shira Dunsiger, Fadilatou Toure, Lauren Connell Bohlen, Tanya J. Benitez, Cara M. Murphy, Dominika M. Pindus, Candace S. Brown, Bess H. Marcus

**Affiliations:** 1 Department of Behavioral and Social Sciences, Brown University School of Public Health, Providence, Rhode Island; 2 Department of Kinesiology, University of Massachusetts Amherst, Amherst, Massachusetts; 3 Department of Epidemiology and Community Health, University of North Carolina at Charlotte, Charlotte, North Carolina

**Keywords:** Physical activity, youth sports, mothers, community-based, pilot

## Abstract

**Introduction:**

Mothers have increased risk for insufficient physical activity and face various barriers to engaging in physical activity. Youth sports have been examined as a setting for health-promotion interventions for children but not for parents. The authors conducted a 6-week single-arm open pilot trial of a physical activity intervention delivered to mothers in Rhode Island, U.S. during children’s sports practices.

**Methods:**

The authors partnered with a youth football organization to deliver the intervention, which consisted of group physical activity sessions during practice. Feasibility was determined on the basis of whether enrollment (i.e., ≥8 participants per week), retention (i.e., ≥80% of participants through 6 weeks), and assessment (i.e., obtaining usable physical activity data from ≥80% of participants) targets were met. Acceptability was determined on the basis of participant responses to a consumer satisfaction questionnaire (with the intervention deemed satisfactory if ≥80% of participants were satisfied with the intervention) and engagement (i.e., intervention session attendance). Preliminary efficacy was determined on the basis of whether participants experienced increases in moderate-to-vigorous physical activity, assessed through 7-day physical activity recall, or physical activity self-efficacy, assessed through the self-efficacy for physical activity measure, at 3 and 6 weeks relative to baseline. The authors descriptively analyzed postintervention feasibility, acceptability, sociodemographic characteristics, and preliminary efficacy outcomes. To explore preliminary efficacy estimates, the authors ran a series of unadjusted paired *t*-tests to compare baseline moderate-to-vigorous physical activity and self-efficacy with moderate-to-vigorous physical activity and self-efficacy at 3 and 6 weeks.

**Results:**

The authors met recruitment (*n*=26) and retention (85%) targets but only collected follow-up data from 12 participants (aged 30–62 years). Twelve of 18 planned intervention sessions were offered owing to practice cancellations; participant attendance ranged from 0 to 12 sessions (mean=3.52, SD=4.07). All participants (100%) were satisfied with the intervention, and significant increases in moderate-to-vigorous physical activity and physical activity self-efficacy were observed. Specifically, an increase in moderate-to-vigorous physical activity from baseline to 3 weeks of 114.5 minutes was observed (Cohen’s d=0.75), whereas self-efficacy scores increased by 0.98 (Cohen’s d=0.97). Self-efficacy scores were increased by 1.07 (Cohen’s d=0.91) at 6 weeks.

**Conclusions:**

This study demonstrated the feasibility of delivering a physical activity intervention to mothers during youth sports practices, although the authors did not reach the assessment target. Participants were satisfied with the intervention, and increases in physical activity outcomes were observed. However, studies examining strategies to increase engagement and maximize benefits, particularly among insufficiently active mothers, would be beneficial. Nonetheless, this is the first study to demonstrate the potential of youth sports practices as a setting for delivering health promotion interventions to parents.

## HIGHLIGHTS

• This is the first study to utilize youth sports for parent health promotion.
• The pilot trial demonstrated feasibility, acceptability, and preliminary efficacy.
• Significantly increases in physical activity and self-efficacy were observed.

## INTRODUCTION

Parenthood is associated with behavior changes that affect health because parents are less physically active than nonparents, with risk for insufficient physical activity (PA) and elevated BMI especially high among mothers.^1^, ^2^, ^3^, ^4^, ^5^, ^6^, ^7^ The transition to parenthood is a critical window,^8^, ^9^, ^10^ given physical and mental health changes due to changes in social roles and increased stress. Because mothers may additionally struggle with retention of gestational weight gain and social pressure to take on the majority of caregiving responsibilities,^4^^,^^11^, ^12^, ^13^ further impacting PA, numerous studies have tested mother-targeted PA interventions during the postpartum period.^14^^,^^15^ However, mothers of older children (i.e., not just babies) also struggle to meet PA guidelines (e.g., 150 minutes of moderate to vigorous PA [MVPA] per week),^16^ with lack of time, social support, childcare, and guilt of taking away from family time commonly cited as PA barriers.^3^^,^^13^^,^^17^, ^18^, ^19^, ^20^ For example, a 2020 study by Limbers et al.^21^ found that <46% of working mothers met the aerobic PA guideline. Thus, innovative PA interventions that target these barriers among mothers of older children are needed.

An estimated 51% of U.S. children aged 6–17 years participate in youth sports, which is expected to increase, given that one of the targets of *Healthy People 2030* is to increase youth sports participation to 63%.^22^ Given how common youth sports participation is and its impact on health (e.g., associations with increased PA and mental health benefits), in 1991, Smith and Smoll declared the sports environment as an “inviting but largely untapped naturalistic laboratory for behavioral research and intervention.”^23^ Today, it is no longer untapped; researchers have studied youth sports as a setting for health promotion among children.^24^, ^25^, ^26^, ^27^, ^28^, ^29^, ^30^ However, what remains unknown is whether youth sports can also be a setting for parent health promotion—until now.

Youth sports exacerbate mothers’ PA time barriers because they often require time commitment from parents. PA time barriers reported by mothers include scheduling conflicts, having too many responsibilities, and prioritizing children’s commitments,^4^^,^^11^, ^12^, ^13^^,^^31^ which are all relevant to youth sports participation. Paradoxically, when parents promote PA among children through sports, their own PA may be negatively impacted. They may find themselves more sedentary as a result of spending time transporting children and/or sitting and watching children engage in sports, especially if they stay for practices. For example, in Rhode Island, youth football leagues generally begin during summer, with most teams holding 2-hour practices 5 days per week. Although such a time commitment can be a barrier to parent PA, it may also provide an opportunity to help parents meet PA guidelines.^16^

To examine the potential of youth sports practices as a setting for delivering parent-targeted PA interventions, the authors partnered with a youth football organization in Providence, RI to pilot test a PA intervention for mothers. The intervention addresses common PA barriers by capitalizing on the time many mothers already spend at their children’s sports practices. Such an approach is responsive to findings from a systematic review by Hartman and colleagues^32^ (2010), which suggested that embedding interventions into the mother and child routine increases effectiveness. The group-based intervention, which aims to foster social support, is also responsive to prior research demonstrating the efficacy of group-based PA interventions for mothers.^33^, ^34^, ^35^ The authors report on the intervention’s feasibility, acceptability, and preliminary efficacy and share insights learned regarding the potential of youth sports as a setting for delivering interventions to promote parents’ PA.

## METHODS

### Study Sample

Eligible participants were aged least 18 years, were female, were mother/primary caregiver of a child who was on 1 of the organization’s football teams or cheerleading squads, had email access, and spoke English. For safety reasons, participants also needed to pass the Physical Activity Readiness Questionnaire.^36^ Participants were recruited by research assistants who distributed flyers at the field; the president of the organization also shared the flyer with parents. Ethical approval for the study was received from Brown University, all participants provided written informed consent, and the study was registered at www.clinicaltrials.govNCT05461742.

The authors followed a staged approach to refine and test the intervention.^37^ First, the authors formed a community advisory board (CAB) of individuals within the partner organization to ensure that the intervention was culturally relevant, feasible, and acceptable to the organization and its families. Represented on the CAB were the organization’s president, vice president, a football coach, and a team mom. Over 5 meetings (3 held in person and 2 through phone, facilitated by the principal investigator), the CAB assisted in intervention design, providing input on intervention content, structure, and implementation. A key example of a revision to the program made by the CAB was around the timing of the intervention sessions. The CAB emphasized the importance of mothers being able to get their child settled at the start of practice and also being available to hear any coach announcements at the end of practice, so sessions were scheduled to begin 30 minutes after practice began and end 30 minutes before practice ended.

The resulting intervention consisted of in-person group PA sessions offered during practice at the outdoor practice field. Three PA sessions were scheduled per week over 6 weeks, free of charge, and participants could attend as desired. All PA equipment used during the sessions, which included resistance bands, kettle bells, jump ropes, and battle ropes, was provided on site at the field, and each participant was given a yoga mat. Each session lasted for 60 minutes and consisted of a workout plan that included activities that emphasize moderate-to-vigorous PA (MVPA) activities. Sessions followed a consistent structure (5-minute warm-up, 50 minutes of circuit-style aerobic and strength training activities with modifications to accommodate different fitness levels, and 5-minute cool down), but the specific exercises and challenges progressed over the 6 weeks to gradually build confidence, fitness, and engagement. Examples of exercises in the main workout included jogging, jumping jacks, burpees, squats, lunges, and push-ups. Sessions were facilitated by a single experienced female personal trainer, who was a mother in the organization and member of the CAB. Child supervision from research assistants during sessions was available for participants with additional children who were not practicing (e.g., those with young children).

The intervention was explicitly guided by Social Cognitive Theory, targeting 3 primary constructs: self-efficacy, observational leaning, and reinforcement.^38^ These constructs informed both the overall program structure and the specific design of each session. For self-efficacy, the intervention aimed to increase participants’ confidence in their ability to engage in regular PA, despite being busy mothers, by demonstrating how they could be active while also being involved in and supportive of their children’s extracurricular activities. The intervention also aimed to increase confidence in ability to perform exercises irrespective of fitness level by offering modifications for all activities, providing verbal encouragement, and using a graduated progression of intensity over the 6 weeks. The personal trainer modeled correct technique and emphasized small wins, such as completing short intervals of jogging or completing more repetitions over time. With respect to observational learning, during demonstrations, the trainer highlighted proper form, safe pacing, and ways to adapt exercises for varying fitness levels. The group-based nature of the exercises, along with the inclusion of women with various fitness levels and baseline PA, also provided opportunity for participants to learn from their peers. Seeing other mothers participate, especially the trainer, who was a fellow parent and member of the organization, helped normalize PA during practice and provided participants with relatable role models. For reinforcement, participants received verbal praise during sessions and public acknowledgment from the organization (e.g., at the end of the year banquet). These strategies were woven into the intervention rather than treated as separate components.

The intervention was piloted during September and October of 2022, when team practices were also scheduled 3 days per week. The authors conducted a 6-week single-arm open pilot trial, with participants completing assessments at baseline, 3 weeks, and 6 weeks. First, participants completed eligibility screening and informed consent procedures. Second, participants scheduled a time to complete the phone portion of the baseline assessment, which consisted of completing a 7-day PA recall (PAR),^39^ and online questionnaire. At 3 and 6 weeks, phone calls and online questionnaires were again administered. Participants received incentives for completing assessments, totaling $125. The session schedule was emailed to participants in advance, and attendance was taken at each session.

### Measures

Feasibility was determined on the basis of whether targets for enrollment (i.e., ≥8 participants per week), retention (i.e., ≥80% of participants through 6 weeks), and assessment (i.e., obtaining usable PA data from ≥80% of participants) were achieved. These thresholds were informed by existing literature on pilot intervention research,^40^^,^^41^ the intervention’s expected effect size in a fully powered RCT given the team’s prior work PA promotion among women,^42^, ^43^, ^44^ and contextual factors specific to the study population and setting (e.g., number of families in the organization).

Acceptability was determined on the basis of participant responses to a consumer satisfaction questionnaire that the team has used in prior studies^42^^,^^45^, ^46^, ^47^ (Table 1). This questionnaire was included in the 6-week assessment. The intervention would be deemed satisfactory if ≥80% of participants were satisfied or very satisfied with the intervention. The authors also examined participant engagement with the intervention by tracking participant session attendance.

**Table 1:** Participant Satisfaction With the Intervention

Survey items	Study sample (*n*=13)	Those who completed multiple 7-day PARs (*n*=11)	Those already meeting guidelines at baseline with multiple 7-day PARs (*n*=3)	Those not meeting guidelines at baseline with multiple 7-day PARs (*n*=8)
How satisfied were you with the program overall? I was not satisfied I was a little satisfied I was satisfied I was very satisfied	0 (0%) 0 (0%) 3 (23%) 10 (77%)	0 (0%) 0 (0%) 2 (18%) 9 (82%)	0 (0%) 0 (0%) 0 (0%) 3 (100%)	0 (0%) 0 (0%) 2 (25%) 6 (75%)
How pleasing did you find the physical activity sessions? They were not pleasing at all A little pleasing They were pleasing They were very pleasing	0 (0%) 2 (15%) 2 (15%) 9 (69%)	0 (0%) 1 (9%) 2 (18%) 8 (73%)	0 (0%) 0 (0%) 0 (0%) 3 (100%)	0 (0%) 1 (13%) 2 (25%) 5 (63%)
How beneficial did you find the physical activity sessions? Not beneficial at all A little beneficial They were beneficial Very beneficial	1 (8%) 0 (0%) 5 (38%) 7 (54%)	1 (9%) 0 (0%) 3 (27%) 7 (64%)	0 (0%) 0 (0%) 1 (33%) 2 (67%)	1 (13%) 0 (0%) 2 (25%) 5 (63%)
Do you think that you learned something about exercising from participating in this program? Yes No	13 (100%) 0 (0%)	11 (100%) 0 (0%)	3 (100%) 0 (0%)	8 (100%) 0 (0%)
Would you recommend the program to a friend? Yes No	13 (100%) 0 (0%)	11 (100%) 0 (0%)	3 (100%) 0 (0%)	8 (100%) 0 (0%)
How motivated do you feel to continue exercising after the program? I do not feel motivated I feel a little motivated I feel motivated I feel very motivated	0 (0%) 3 (23%) 5 (38%) 5 (38%)	0 (0%) 2 (18%) 4 (36%) 5 (45%)	0 (0%) 1 (33%) 1 (33%) 1 (33%)	0 (0%) 1 (13%) 3 (38%) 4 (50%)

*Note: n* (%) are presented.

Preliminary efficacy was determined on the basis of whether participants experienced changes in weekly minutes of MVPA or PA self-efficacy. Weekly minutes of MVPA were assessed using the 7-day PAR^39^ at each assessment. The 7-day PAR has consistently demonstrated acceptable reliability, internal consistency, and congruent validity with objective PA measures,^48^, ^49^, ^50^, ^51^ along with sensitivity to changes in MVPA.^52^ PA self-efficacy was assessed using the self-efficacy for PA measure,^53^ which has high internal consistency, and was included in the online questionnaire at each assessment.

Sociodemographic characteristics were assessed at baseline through the online questionnaire and included age, number of children living in the house, marital status, employment status, educational attainment, annual household income, race, and ethnicity. Participants were allowed to select all that apply for race, and their race/ethnicity was classified using the Department of Finance method.^54^ Finally, participants self-reported their height and weight, which was used to calculate their BMI.

### Statistical Analysis

The authors descriptively analyzed postintervention feasibility, acceptability, sociodemographic characteristics, and preliminary efficacy outcomes. Because the primary aim of pilot studies was to assess feasibility and acceptability, the authors did not calculate power to detect efficacy. However, the authors examined within-person changes over time in weekly minutes of MVPA and PA self-efficacy to explore preliminary efficacy estimates. The authors first ran a series of unadjusted paired *t*-tests to compare baseline MVPA and self-efficacy with MVPA and self-efficacy at 3 and 6 weeks. The authors then conducted a stratified analysis to examine the same changes separately for participants who were already meeting PA guidelines at baseline (i.e., self-reported ≥150 minutes of weekly MVPA) and participants who were not meeting PA guidelines at baseline. Analyses were performed using StataSE statistical software.

## RESULTS

In terms of feasibility, the authors successfully recruited 26 participants between August 24, 2022 and September 13, 2022, which surpassed the target of ≥8 participants per week. Participants were on average aged 39 years (SD=9.4) and had 2.0 children (SD=1.37), with 84% of participants classified as overweight or obese on the basis of their BMI (i.e., had a BMI ≥25). A third of participants were Hispanic, 47% were Black, 7% were American Indian/Alaska Native, and 13% were White. The sample was diverse with respect to marital status and SES (Table 2). Four participants withdrew from the study, resulting in a retention rate of 85%, surpassing the 80% target. However, although 7-day PAR data were collected for 21 participants at baseline, they were only collected for 10 participants at 3 weeks and 11 participants at 6 weeks. Because the analyses for assessing preliminary efficacy included only participants from whom the authors had 7-day PAR data for at least 2 time points, the analytical sample is thus 12. Thus, the authors did not meet the assessment completion target. Women who only completed the baseline assessment (none of whom were meeting PA guidelines) were more likely to be married and had higher levels of education and annual household incomes than those in the analytical sample; they also attended fewer PA sessions.

**Table 2:** Sample Sociodemographic Characteristics

Characteristic	Study sample (*n*=22)	Those who completed multiple 7-day PARs (n=12)	Those already meeting guidelines at baseline with multiple 7-day PARs (*n*=4)	Those not meeting guidelines at baseline with multiple 7-day PARs (*n*=8)
Age, years Missing (*n*)	39.08 (9.40) 10	40.14 (12.43) 5	44.00 (12.73) 2	38.60 (13.45) 3
Number of children living in house Missing (*n*)	2.00 (1.37) 6	1.90 (1.10) 2	2.50 (1.00) 0	1.50 (1.05) 2
Marital status Never married Divorced Separated Widowed Married Living with partner Missing (*n*)	7 (41%) 0 (0%) 1 (6%) 2 (12%) 6 (35%) 1 (6%) 5	6 (60%) 0 (0%) 1 (10%) 2 (20%) 0 (0%) 1 (10%) 2	2 (50%) 0 (0%) 0 (0%) 1 (25%) 0 (0%) 1 (25%) 0	4 (67%) 0 (0%) 1 (17%) 1 (17%) 0 (0%) 0 (0%) 2
Employment status Unemployed Full time Part time Missing (*n*)	0 (0%) 9 (56%) 7 (44%) 6	0 (0%) 5 (50%) 5 (50%) 2	0 (%) 3 (75%) 1 (25%) 0	0 (%) 2 (33%) 4 (67%) 2
Race/ethnicity Black Hispanic American Indian/Alaskan Native White Missing (*n*)	7 (47%) 5 (33%) 1 (7%) 2 (13%) 7	5 (50%) 3 (30%) 1 (10%) 1 (10%) 2	2 (50%) 1 (25%) 1 (25%) 0 (0%) 0	3 (50%) 2 (33%) 0 (0%) 1 (17%) 2
Educational attainment <12 years High school Vocational/technical school Some college College graduate Missing (*n*)	1 (6%) 3 (19%) 3 (19%) 5 (31%) 4 (25%) 6	0 (0%) 3 (30%) 3 (30%) 3 (30%) 1 (10%) 2	0 (0%) 1 (25%) 1 (25%) 2 (50%) 0 (0%) 0	0 (0%) 2 (33%) 2 (33%) 1 (17%) 1 (17%) 2
Annual household income <$10k $10k≤20k $20k≤30k $30k≤40k $40k≤50k ≥$50k Missing (*n*)	2 (12%) 2 (12%) 1 (6%) 2 (12%) 4 (24%) 6 (35%) 5	2 (20%) 2 (20%) 0 (0%) 1 (10%) 3 (30%) 2 (20%) 2	0 (0%) 1 (25%) 0 (0%) 0 (0%) 2 (50%) 1 (25%) 0	2 (33%) 1 (17%) 0 (0%) 1 (17%) 1 (17%) 1 (17%) 2
BMI <18.5 18.5–24.9 25–29.9 ≥30 Missing (*n*)	0 (0%) 2 (15%) 5 (38%) 6 (46%) 9	0 (0%) 2 (25%) 2 (25%) 4 (50%) 4	0 (0%) 1 (33%) 0 (0%) 2 (67%) 1	0 (0%) 1 (20%) 2 (40%) 2 (40%) 3
Intervention sessions attended Missing (*n*)	3.52 (4.07) 0	5.00 (4.49) 0	4.25 (3.86) 0	5.38 (4.98) 0

*Note*: Mean (SD) are presented for continuous variables; *n* (%) are presented for categorical variables

PAR, physical activity recall.

In terms of acceptability, thirteen participants completed the consumer satisfaction survey (Table 1). All (100%) were satisfied with the intervention, with 77% very satisfied, which exceeded the target. Most participants found the PA sessions pleasing and beneficial, with all participants reporting that they learned something about exercising and saying that they would recommend the program to a friend. When asked how motivated they felt to continue to exercise after the program, 76% felt motivated or very motivated.

In assessing engagement with the intervention through attendance, it should be noted that only 12 of the 18 planned PA sessions were offered owing to organization practice cancellations. Participant attendance ranged from 0 to 12 sessions, with the average number of sessions attended being 3.5 (SD=4.1); 36% of participants attended more than half (i.e., ≥7) of the sessions.

Across the analytical sample, participants reported engaging in an average of 88.17 (SD=80.46) minutes of MVPA per week at baseline, 205.30 (SD=141.42) minutes of MVPA at 3 weeks, and 100.45 (SD=75.91) minutes of MVPA at 6 weeks (Table 3). The increase in MVPA from baseline to 3 weeks among the analytical sample was statistically significant at *p*<0.05 (mean change=114.5 minutes, Cohen’s d=0.75). Participants already meeting the PA guidelines at baseline reported engaging in an average of 180.75 (SD=16.09) minutes of MVPA per week at baseline, 219.50 (SD=130.73) minutes of MVPA at 3 weeks, and 96.67 (SD=70.24) minutes of MVPA at 6 weeks. Participants not meeting the PA guidelines at baseline reported engaging in an average of 41.88 (SD=52.09) minutes of MVPA per week at baseline, 195.83 (SD=159.61) minutes of MVPA at 3 weeks, and 101.88 (SD=82.55) minutes of MVPA at 6 weeks. The increase in MVPA from baseline to 3 weeks among participants not meeting PA guidelines at baseline was statistically significant at *p*<0.05 (mean change=165 minutes, Cohen’s d=1.06).

**Table 3:** Self-Reported Weekly Minutes of MVPA at Baseline, 3 Weeks, and 6 Weeks

Sample	Baseline mean (SD) (95% CI)	3-weeks mean (SD) (95% CI)	*p*-value for baseline to 3-weeks comparison (*n*)	6-weeks mean (SD) (95% CI)	*p*-value for baseline to 60-week comparison (*n*)
Analytical sample	88.17 (80.46) (37.05, 139.29)	205.30 (141.42) (104.14, 306.46)	0.041* (*n*=10)	100.45 (75.91) (49.46, 151.45)	0.556 (*n*=11)
Those already meeting guidelines at baseline	180.75 (16.09) (155.15, 206.35)	219.50 (130.73) (11.48, 427.52)	0.585 (*n*=4)	96.67 (70.24) (−77.81, 271.15)	0.123 (*n*=3)
Those not meeting guidelines at baseline	41.88 (52.09) (−1.68, 85.43)	195.83 (159.61) (28.34, 363.33)	0.049* (*n*=6)	101.88 (82.55) (32.86, 170.89)	0.144 (*n*=8)

*Note*: Boldface indicates statistical significance (*p<0.05).

MVPA, moderate-to-vigorous physical activity.

Across the analytical sample, the average self-efficacy score at baseline was 2.13 (SD=0.64), whereas at 3 weeks, it was 2.93 (SD=1.03), and at 6 weeks, it was 3.24 (SD=1.17), with higher scores indicating greater confidence for engaging in PA (Table 4). The increase in self-efficacy from baseline to 3 weeks (mean change=0.98, Cohen’s d=0.97) and baseline to 6 weeks (mean change=1.07, Cohen’s d=0.91) among the analytical sample was statistically significant at *p*<0.05. Among participants already meeting the PA guidelines at baseline, the average self-efficacy score at baseline was 2.33 (SD=0.50), whereas at 3 weeks, it was 4.00 (SD=0.85), and at 6 weeks, it was 3.73 (SD=1.14). The increase in self-efficacy from baseline to 3 weeks among participants meeting PA guidelines at baseline was statistically significant at *p*<0.05 (mean change=1.70, Cohen’s d=12.02). Among participants not meeting the PA guidelines at baseline, the average self-efficacy score at baseline was 2.03 (SD=0.72), whereas at 3 weeks, it was 2.77 (SD=0.90), and t 6 weeks, it was 3.03 (SD=1.21).

**Table 4:** Self-Efficacy for Physical Activity at Baseline, 3 Weeks, and 6 Weeks

Sample	Baseline mean (SD) (95% CI)	3 weeks mean (SD) (95% CI)	*p*-value for baseline to 3-week comparison (*n*)	6-week mean (SD) (95% CI)	*p*-value for baseline to 6-week comparison (*n*)
Analytical sample	2.13 (0.64) (1.64, 2.63)	2.93 (1.03) (2.14, 3.72)	0.029* (*n*=8)	3.24 (1.17) (2.40, 4.08)	0.026* (*n*=9)
Those already meeting guidelines at baseline	2.33 (0.50) (1.08, 3.58)	4.00 (0.85) (−3.62, 11.62)	0.037* (*n*=2)	3.73 (1.14) (0.91, 6.56)	0.118 (*n*=3)
Those not meeting guidelines at baseline	2.03 (0.72) (1.28, 2.79)	2.77 (0.90) (1.82, 3.71)	0.152 (*n*=6)	3.03 (1.21) (1.91, 4.15)	0.159 (*n*=6)

*Note*: Boldface indicates statistical significance (*p<0.05).

## DISCUSSION

The findings regarding high recruitment and retention rates demonstrate the feasibility of a PA intervention delivered to mothers during youth sports practices. Although session attendance varied across the sample, participants were highly satisfied with the intervention, significant increases in self-reported minutes per week of MVPA and PA self-efficacy were observed, and no adverse events were reported. However, missing data were abundant because many participants did not complete assessments. Overall, the intervention shows promise but would benefit from efforts to increase engagement and assessment completion.

The demonstrated feasibility and acceptability are likely the result of it being designed specifically to address the PA barriers mothers face. Delivering the intervention during youth sports practices allows mothers to simultaneously support their children’s extracurricular activities while engaging in PA, addressing time and guilt-related barriers. Provided child supervision addresses childcare barriers. The sessions’ group-based nature allows mothers to connect with and engage in PA with other mothers from their children’s sports organization, which can foster social support. Furthermore, delivering the intervention free of charge at a location mothers already travel to addresses cost and transportation barriers.^55^, ^56^, ^57^, ^58^ Notably, this sample was composed of predominantly racial or ethnic minority women, and intervention engagement was highest among single mothers and those with lower levels of education and income. The intervention aimed to make engaging in PA as easy as possible for busy mothers, and the authors had no issues recruiting participants, all of whom said that they would recommend the program to a friend.

However, session attendance varied widely across participants. Undoubtedly, this was largely influenced by the organization with whom the authors partnered allowing the coaches of each team to determine their practice schedule once school began. Although each team practiced 3 days per week, overlap with PA session days varied across teams. PA sessions were scheduled for Tuesdays, Thursdays, and Saturdays, which mirrored at least 1 team’s practice schedule, whereas other teams practiced on Mondays, Wednesdays, and/or Fridays. Future implementations should aim to offer sessions on all practice days. Another strategy to optimize dose delivery that could be considered in future implementations is to offer alternates to the in-person sessions. This could include virtual or hybrid sessions, which would help mitigate the impact of cancelled practices. The authors also observed that as the season progressed, more parents began dropping their children off and picking them up than staying at practice. Parents may be forced to choose between using practice to be physically active or attending to other responsibilities. Alternatively, parents may find that practice time is the only downtime that they have and prefer to use it to relax. As such, another future direction could be to provide resources to support asynchronous workouts. This could include written workout plans or videos and providing mothers with their own PA equipment to allow for greater flexibility, enhancing accessibility and potentially adherence. Finally, future studies should assess whether a dose–response relationship exists because this could inform the necessary minimum effective dose for the intervention to achieve a meaningful increase in PA.

Despite not all participants engaging with the intervention, the authors observed significant increases in self-reported minutes per week of MVPA, especially among mothers who were not meeting PA guidelines at baseline. These mothers significantly increased their MVPA from baseline to 3 weeks; MVPA at 6 weeks was also higher than at baseline but not significantly. Notably, the observed increase in average reported MVPA from baseline to each time point was clinically meaningful. However, the findings suggest that improvements to maintain engagement after the first few weeks may be beneficial. That said, it is worth noting that a third of the intervention PA sessions were canceled owing to inclement weather, and cancellations occurred more frequently later in the season. This is consistent with prior research demonstrating weather-related seasonality effects on PA,^59^ and although not significant, the authors also observed that MVPA decreased among the mothers who were meeting PA guidelines at baseline later in the season. Future iterations should consider PA promotion during inclement weather. Notably, the aforementioned personal PA equipment, hybrid, and asynchronous workouts, particularly if designed as indoor/home workouts, may serve as a buffer to weather disruptions.

The authors also observed significant increases in PA self-efficacy. Significant increases were observed among the analytical sample as well as among mothers who were already meeting PA guidelines at baseline. Typically, such mothers are not eligible to participate in PA interventions. However, owing to the community-based nature of this intervention, the authors did not want to exclude active mothers who may provide social support to underactive mothers. That the mothers who were already meeting PA guidelines experienced significant increases in PA self-efficacy at 3 weeks shows that they can still benefit from PA promotion interventions. This intervention may have inspired active mothers to better utilize their time, thereby increasing their self-efficacy for PA. Although not assessed, if active mothers begin meeting their PA needs during practice as a result of such interventions, it could free up some time outside of practice (e.g., by not having to go to the gym), which could lead to additional benefits.

### Limitations

This intervention required partnering with sports organizations and thus relinquishing some control around intervention dose and delivery. Future studies would benefit from studying the core components and functions of interventions tested for efficacy in youth sports settings. Researchers can leverage implementation science methods to track fidelity and adaptations to inform implementation strategies, which preserve efficacy across settings. Similarly, future studies might consider evaluating the role of intervention context (i.e., practice location), content/dose, and mode of delivery as moderators of effect size to determine the potential role of each characteristic from both an efficacy and future implementation perspective.

Second, this single-arm open pilot trial did not include a control group, and many participants did not complete the assessments, resulting in missing data. As such, although the primary aim was to demonstrate feasibility and acceptability, caution is warranted when interpreting efficacy findings. Because the absence of a control group makes it difficult to attribute the observed changes in MVPA and self-efficacy to the intervention, future studies should utilize randomized controlled designs. The nature and setting of this intervention make it particularly well suited for a cluster-randomized trial with a waitlist control. Importantly, although it is typical for sufficiently active individuals to be ineligible for PA promotion interventions, the authors did not exclude such individuals from this study because it was important to the CAB that the program be offered more broadly, and the authors did not create an impression that it was only for unfit mothers. However, this could result in a ceiling effect, biasing results toward the null. Regarding the assessments, the authors received feedback from participants that they were too repetitive and close together, and participants did not like that they had to complete the 7-day PAR over the phone. Future studies might benefit from utilizing other PA measures (e.g., accelerometry, which notably is not subject to social desirability or recall bias the way self-reported PA is) or assessment methods (e.g., in-person 7-day PAR), with consideration for the length and timing of assessments. Additional strategies that may minimize missing data include having more flexible assessment windows, sending reminders through text messages, and utilizing in-person follow-up. Nonetheless, this study provides preliminary data for future studies utilizing stronger study designs (e.g., a fully powered cluster RCT with multiple sports organizations).

## CONCLUSIONS

This is the first study to demonstrate the potential of youth sports practices as a setting for delivering health promotion interventions to parents. Findings demonstrated the feasibility, acceptability, and preliminary efficacy of a PA-promotion intervention delivered to mothers during youth sports practices. Participants, irrespective of their level of PA at baseline, benefited from the intervention. However, future studies should examine strategies to increase participant engagement with the intervention and maximize PA benefits, particularly among insufficiently active mothers. Future studies, including fully powered RCTs, could also assist in further strengthening the intervention content and structure and informing implementation strategies to maximize effectiveness. Given that more than half of children participate in sports, interventions delivered in this setting have the potential for widespread reach to positively impact parent PA and other health behaviors.

## CRediT authorship contribution statement

**Tayla :** Conceptualization, Funding acquisition, Data curation, Methodology, Formal analysis, Writing – original draft, Writing – review & editing. **Belinda O’Hagan:** Visualization, Writing – original draft, Writing – review & editing. **Sugandha K. Gupta-Louis:** Data curation, Writing – original draft, Writing – review & editing. **Shira Dunsiger:** Methodology, Formal analysis, Writing – review & editing. **Fadilatou Toure:** Investigation, Writing – review & editing. **Lauren Connell Bohlen:** Writing – review & editing. **Tanya J. Benitez:** Writing – review & editing. **Cara M. Murphy:** Writing – review & editing. **Dominika M. Pindus:** Writing – review & editing. **Candace S. Brown:** Writing – review & editing. **Bess H. Marcus:** Conceptualization, Funding acquisition, Supervision, Writing – review & editing.
